# Cost effectiveness of an intervention focused on reducing bathing disability

**DOI:** 10.1007/s10433-016-0404-1

**Published:** 2016-11-28

**Authors:** Magnus Zingmark, Ingeborg Nilsson, Fredrik Norström, Klas Göran Sahlén, Lars Lindholm

**Affiliations:** 1 Division of Occupational Therapy, Department of Community Medicine and Rehabilitation, Umeå University, Umeå, Sweden; 2 Graduate School in Population Dynamics and Public Policy, Umeå University, Umeå, Sweden; 3 ALC (Ageing and Living Conditions), Umeå University, Umeå, Sweden; 4 Epidemiology and Public Health, Umeå University, 90187 Umeå, Sweden; 5 Department of Nursing, Umeå University, Umeå, Sweden; 6 Community Care Administration, Municipality of Östersund, 83182 Östersund, Sweden

**Keywords:** Cost effectiveness, QALY, Occupational therapy intervention, Reablement

## Abstract

The onset of bathing disability among older people is critical for a decline in functioning and has implications for both the individuals’ quality of life and societal costs. The aim of this study was to evaluate long-term cost effectiveness of an intervention targeting bathing disability among older people. For hypothetical cohorts of community-dwelling older people with bathing disability, transitions between states of dependency and death were modelled over 8 years including societal costs. A five-state Markov model based on states of dependency was used to evaluate Quality-adjusted life years (QALYs) and costs from a societal perspective. An intervention group was compared with a no intervention control group. The intervention focused on promoting safe and independent performance of bathing-related tasks. The intervention effect, based on previously published trials, was applied in the model as a 1.4 increased probability of recovery during the first year. Over the full follow-up period, the intervention resulted in QALY gains and reduced societal cost. After 8 years, the intervention resulted in 0.052 QALYs gained and reduced societal costs by €2410 per person. In comparison to the intervention cost, the intervention effect was a more important factor for the magnitude of QALY gains and long-term societal costs. The intervention cost had only minor impact on societal costs. The conclusion was that an intervention targeting bathing disability among older people presents a cost-effective use of resources and leads to both QALY gains and reduced societal costs over 8 years.

## Introduction

Bathing disability is common among people older than 80 years (Jagger et al. 2001; Naik et al. 2004) and is associated with a high risk of disability in other activities of daily living (ADL) (Gill et al. 2006b; Jagger et al. 2001), the amount of informal and formal help (LaPlante et al. 2002), admission to a nursing home (Gill et al. 2006a) and death (Rozzini et al. 2007). Bathing is defined as washing and drying one’s entire body (Naik et al. 2004; World Health Organization 2002), and disability in bathing has been defined as experiencing difficulty in performing the activity (Jagger et al. 2001) or being dependent (Gill et al. 2006b). Bathing is a complex activity, including several subtasks that are to be performed in a demanding environment (e.g. wet floors) challenging a person’s physical and cognitive skills (Naik et al. 2004). The ability to bathe independently is important to achieve a sense of well-being and to fulfil social expectations. Older people who are independent in bathing have strong preferences to remain independent (Ahluwalia et al. 2010; Vik et al. 2007) but anticipate bathing to become a future problem threatening their independence (Ahluwalia et al. 2010). The transition from being independent to becoming dependent on help from spouses, friends or the community in daily living is detrimental to quality of life (QoL) (Hellström et al. 2004; Johannesen et al. 2004) and has a significant impact on societal costs (Lindholm et al. 2013).

Previous studies have indicated that rehabilitative interventions, including a few home visits, targeting older people with bathing disability have short-term effects on improving the ability to bathe and reduce dependency on home help in bathing (Chiu and Man 2004; Zingmark and Bernspång 2011). The results from a recent trial show that an intervention implemented to support independence in activities of daily living (ADL) for older people referred for problems with personal care (including bathing) had significant effects on reduced dependency for as long as up to 2 years (Lewin et al. 2013a, 2014). Although the existing evidence indicates that costs for health and homecare can be reduced as a result of an intervention promoting independence among older people (Cook et al. 2013; Lewin et al. 2013b), no trial has evaluated cost effectiveness in terms of costs per quality-adjusted life years (QALYs) gained. Because the identification of cost-effective interventions is important in terms of deciding which interventions to implement (Broqvist et al. 2011), there is a need to evaluate the cost effectiveness of an intervention targeting older people with bathing disability. An important aspect in investigating cost effectiveness is to consider the time horizon for which both the effects, in terms of QALYs, as well as costs are evaluated. Preferably, a lifetime perspective should be adopted (Drummond et al. 2005). By the use of decision modelling (Briggs et al. 2006; Drummond et al. 2005), we used existing evidence from clinical trials (Chiu and Man 2004; Lewin et al. 2013a, 2014; Zingmark and Bernspång 2011) to extrapolate cost effectiveness over the long term.

The aim of this study was to evaluate the cost effectiveness of an intervention implemented to minimize bathing disability for older people with bathing disability.

## Method

To evaluate cost effectiveness of an intervention targeting community-dwelling older people with bathing disability in comparison to no intervention, we developed a Markov model in Microsoft^®^ Excel 2007. A hypothetical cohort of community-dwelling older people with bathing disability was followed over 8 years with consideration of societal costs in terms of formal health and social care, and informal care. Each state in the Markov model was assigned a score for QoL and a societal cost (including health care, home care, informal care and special accommodation, e.g. nursing home) to allow analysis of long-term cost effectiveness. The design and reporting of the trial were based on the Consolidated Health Economic Evaluation Reporting Standards (CHEERS) statement (Husereau et al. 2013).

## Model structure

**Fig. 1:**
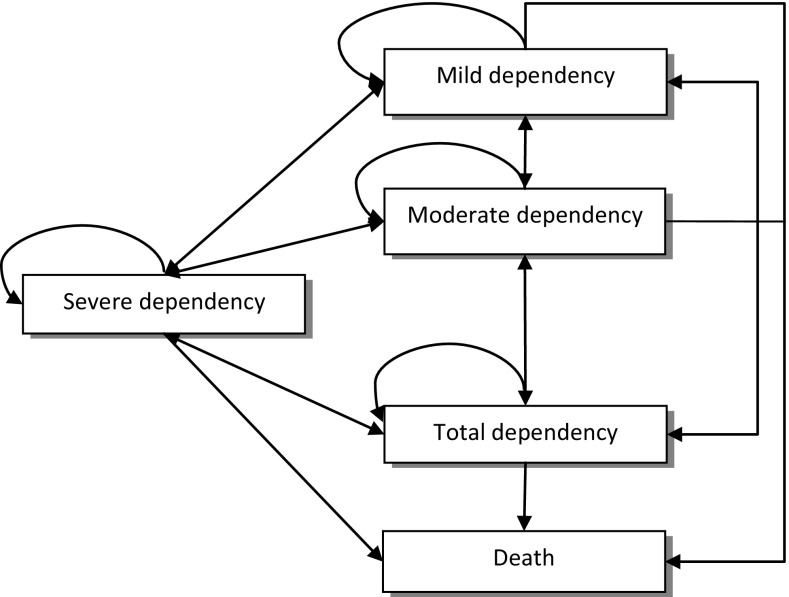
Markov model of transitions between states of dependency and death. Each *arrow* represents a possible transition (i.e. recovery, stability or decline) between two states over a 1-year cycle. *Mild dependency* refers to a state in which a person is independent in personal activities of daily living (PADL), is dependent in a single instrumental activity of daily living (IADL) and needs help no more than one time per week. *Moderate dependency* refers to a state in which a person is independent in PADL, is regularly dependent in more than one IADL and needs help more than one time per week. S*evere dependency* refers to a state in which a person is dependent in one PADL and more than one IADL and needs help one or several times per day. *Total dependency* refers to a state in which a person is dependent in at least one PADL and IADLs and needs help one or several times per day and lives at a special housing

In close collaboration with a group of experienced social workers, we sought to establish a comprehensive model, with clinically relevant and well-defined states that accurately represented various levels of dependency among older people. The model is in line with earlier research indicating a hierarchy in relation to how dependency develops in activities in daily living (ADL) (Jagger et al. 2001). The states in the model were based on the levels of dependency and place of residency because it has been found that these factors impact self-rated health as well as costs related to health and social care (Lindholm et al. 2013). As a result of the collaboration with the group of social workers, five states were identified: *Mild dependency* refers to a state in which a person is independent in personal activities of daily living (PADL) (e.g. bathing, dressing), is dependent in no more than a single instrumental activity of daily living (IADL) (e.g. cleaning, shopping) and needs help no more than one time per week. *Moderate dependency* refers to a state in which a person is independent in PADL, is regularly dependent in more than one IADL and needs help more than one time per week. S*evere dependency* refers to a state in which a person is dependent in at least one PADL and more than one IADL and needs help one or several times per day. *Total dependency* refers to a state in which a person is dependent in PADLs and IADLs, needs extensive help throughout the day and lives in ordinary or special housing. The final state was death (Fig. 1). Although the model overall illustrates a process towards increasing disability, it is well known that disability among older people involves both decline and recovery (Hardy and Gill 2004). In our study, bathing disability was defined as being dependent (Gill et al. 2006b) of help from another person with bathing, in our model represented by the state severe dependency. All participants started in the severe dependency state. The cycle in the model was one year. Figure 1 displays possible transitions, i.e. recovery to a less dependent state or decline to a more dependent state or death.

## Transition probabilities

In order to extrapolate transitions in the levels of dependency in a cohort of older people, the best longitudinal data we identified were from a Canadian study by Raîche et al. including participants 75 years or older from the general population who were identified as being at risk for functional decline (Raîche et al. 2012). Based on Iso-SMAF (SMAF is a French acronym for Functional Autonomy Measurement System), a Canadian classification system for disability, including 14 disability profiles (Dubuc et al. 2006), transition probabilities for recovery, stability and decline, was calculated in a cohort of 1410 persons aged 75 years or older at risk for decline in functioning (Raîche et al. 2012). The cohort was followed for 4 years, and annual transition probabilities, including recovery, stability and decline, were estimated. Using the originally reported transition probabilities, we recalculated transition probabilities for our five-state Markov model (Table 1). In our model, the mild dependency state was equivalent to disability profile 1; moderate dependency was equivalent to disability profiles 2–5; severe dependency was equivalent to disability profiles 6–9; and total dependency was equivalent to disability profiles 10–14 + LTCF (Long-Term Care Facility). The probability for a transition was calculated as the sum of probabilities for those profiles, e.g. the probability for a transition from mild to moderate dependency was the sum of probabilities for transitions to disability profiles 2–5. Model parameters (probabilities for transitions between states, QoL scores and societal costs for each state) were obtained from previously published research and are shown in Tables 1 and 2.

**Table 1:** Transition probabilities for annual transitions between states of dependency^a^

	Mild dependency	Moderate dependency	Severe dependency	Total dependency	Death
Mild dependency	**0.79**	0.13	0.03	0.02	0.03
Moderate dependency	0.08	**0.82**	0.03	0.01	0.06
Severe dependency	0.02	0.12	**0.61**	0.11	0.14
Total dependency	0.00	0.03	0.18	**0.63**	0.16
Death					**1.00**

Bold values indicate stability in a state over a one-year period

^a^An example of the probability of a transition over a 1-year period is that a person in the severe dependency state has a probability of 0.12 to recover to the moderate dependency state and a probability of 0.61 to remain stable in the severe dependency state

**Table 2:** Description of each dependency state in our model and correspondence to the Iso-SMAF profiles^a^

	Level of dependency	Iso-SMAF profiles
Mild dependency	Independence in personal activities of daily living (PADL) (e.g. bathing, dressing). Dependence in no more than a single instrumental activity of daily living (IADL) (e.g. cleaning, shopping). Needs help no more than one time per week	Profile 1 includes independence in ADL and having difficulties with IADL
Moderate dependency	Independence in PADL. Regularly dependent in more than one IADL. Needs help more than one time per week	Profiles 2–5 include levels of dependency ranging from a need for supervision in IADL (profile 2) to a need for supervision in ADL and dependency in IADL (profile 5)
Severe dependency	Dependent in at least one PADL and more than one IADL. Needs help one or several times per day	Profiles 6–9 include levels of dependency ranging from difficulties in ADL and dependency in IADL (profile 6) to a need for help in ADL and dependency in IADL (profile 9)
Total dependency	Dependent in PADLs and IADLs. Needs extensive help throughout the day and live in ordinary or special housing	Profiles 10–14 +LTCF^b^ include levels of dependency ranging from extensive need for help in ADL and dependency in IADL to complete dependency. All profiles include severe cognitive impairment

^a^Iso-SMAF profiles (Raîche et al. 2012). (SMAF is a French acronym for Functional Autonomy Measurement System)

^b^Long-Term Care Facility

### Quality of life

Previous studies indicate that a decline in ADL (Fusco et al. 2012) and loss of independence (Andersen et al. 2004; Shearer et al. 2012) has a negative impact on QoL. We searched the literature to assign each state in our Markov model an approximate score for QoL on a scale ranging from 0 to 1 (Drummond et al. 2005). For the state *mild dependency*, we used unpublished baseline data from an ongoing trial including 177 well older people (Zingmark et al. 2014). For the state *moderate dependency*, we made an approximation reflecting a decline in ADL and IADL (Fusco et al. 2012; Szanton et al. 2011). Based on previously published data on decrements in QoL due to major loss of independence (Andersen et al. 2004) and move to a nursing home (Andersen et al. 2004; Honkanen et al. 2006), we approximated QoL scores for the states *severe dependency* and *total dependency*, respectively (Table 2). The QoL scores were multiplied by the time spent in each health state to derive a quality-adjusted life year (QALY) (Drummond et al. 2005). For example, if a person recovers from severe to moderate dependency, the resulting effect over one year will be 0.13 QALYs (from 0.47 to 0.60).

### Societal Cost

To estimate costs for each state, we used data from a Swedish cohort study demonstrating that the levels of dependency in ADL and IADL have a strong impact on total costs (Lindholm et al. 2013) (Table 2). Societal costs are given in Euro (€) and include costs for health care, home help, informal care (assistance or supervision by informal caregiver) and special accommodation.

### Intervention effect

The intervention modelled included rehabilitation that focused on improving a person’s ability to perform self-care tasks related to bathing. Based on previous trials, the content of the intervention included practical training sessions in the person’s home in which therapists focused on encouraging the person to gradually increase her/his ability and self-efficacy to perform bathing-related tasks (Lewin et al. 2013a; Zingmark and Bernspång 2011). The intervention also included the provision of technical aids if deemed necessary. Based on previous trials, we estimated an intervention effect and two alternative intervention costs for (a) an occupational therapy intervention (Zingmark and Bernspång 2011) and (b) a multi-professional intervention (Lewin et al. 2013a). Based on previous trials presented (Table 3), we concluded that the interventions had an effect on recovery from bathing disability in terms of reduced dependency of home care. Although the three studies demonstrated a twofold or higher increased chance of recovery at three months (Chiu and Man 2004; Lewin et al. 2013a; Zingmark and Bernspång 2011), the intervention effect was reduced at one year (Lewin et al. 2013a) to a level that was sustained until two years (Lewin et al. 2014). Therefore, **i**n our model, we applied an intervention effect of 1.4 (Table 3), indicating that the intervention increased the probability of recovery from severe dependency to moderate dependency by 1.4 after one year (i.e. instead of a 12% probability of recovery (Table 1), the intervention increased the probability to 17%). The effect of the intervention was implemented in the analysis as a one-time effect during the first year.

**Table 3:** Estimates of annual costs (€), including health care, home care, informal care and accommodation^a^, and QoL scores Quality of Life ^b^ for each state in the Markov model

Markov state	QoL scores	Total costs
Mild dependency	0.77	2864
Moderate dependency	0.60	8593
Severe dependency	0.47	22,915
Total dependency	0.41	68,746
Dead	0	0

^a^Societal costs (Lindholm et al. 2013)

^b^Quality of Life (QoL) scores (Zingmark et al. 2014; Fusco et al. 2012; Szanton et al. 2011; Andersen et al. 2004)

### Intervention cost

The intervention cost included salaries and the cost for technical aids. The main analysis was based on an occupational therapy intervention (Zingmark and Bernspång 2011) in which it was hypothesized that the intervention on average included 3 home visits for a total of 2 h (Zingmark and Bernspång 2011), which is similar to other trials (Chiu and Man 2004; Gitlin et al. 1999). An alternative intervention cost (used for the sensitivity analysis) was based on a multi-professional (i.e. an occupational therapist, a physiotherapist and a nurse) intervention (Lewin et al. 2013a) including home visits implemented over a time period of 12 weeks. We hypothesized that on average this intervention included 12 home visits. For both the occupational therapy intervention and multi-professional intervention, we approximated the time for travel to home visits and administration to 30 min per home visit. Salaries were based on the gross mean income for occupational therapists in Sweden 28.5 €/h. Costs for technical aids were estimated to be 26 € per person (Zingmark and Bernspång 2011). Based on these figures, the average cost for the occupational therapy intervention was 128 €, and the average cost for the multi-professional intervention was 546 €.

### Statistical analysis

We applied Microsoft^®^ Excel software (Menn and Holle 2009) to analyse the Markov model. Based on previous trials targeting older people with bathing disability (Lewin et al. 2013a; Zingmark and Bernspång 2011), the mean age in the hypothetical cohort was 82 years. The average life expectancy at 82 years, derived from Statistics Sweden, was 8 years for women. Therefore, the analysis included a time period of 8 years. However, it should be noted that men have shorter life expectancy than women. In health economics, it is assumed that people in general have positive time preferences, meaning that the value attached to events that occur in the future is lower than the value attached to identical events in present time. The technique used to handle time preference is called “discounting” (Drummond et al. 2005). QALY scores and societal costs were discounted, i.e. valued lower, at 3% for each year after the first year. For the two alternatives (i.e. intervention vs. no intervention), we calculated the accumulated QALYs and societal costs over 8 years. The main analysis included the cost for the occupational therapy intervention. Results were interpreted in relation to established thresholds indicating a cost ≤11,000 € as a low cost/QALY, a cost ≤55,000 € as a moderate cost/QALY and a cost **>**55,000 € as a high cost/QALY (The National Board of Health and Welfare 2011).

### Sensitivity analysis

To acknowledge uncertainty in parameter estimates, we conducted a sensitivity analysis. Firstly, we hypothesized that a reduced intervention effect and an increased intervention cost would reflect real-world variation that could affect cost effectiveness. Instead of a 1.4 increase in the probability of recovery from severe dependency to moderate dependency as an effect of the intervention, as assumed in the main analysis, we assumed a 1.2 increase of recovery in the sensitivity analysis. Secondly, we assumed that the intervention cost was higher reflecting the multi-professional intervention. We performed the analysis for each of the assumptions separately and both assumptions combined.

## Results

In hypothetical cohorts of 100 people in each group, 17 in the intervention group and 12 in the control group recovered to moderate dependency by the end of the first year as a result of the intervention. The intervention had no direct impact on transitions after the first year. All transition probabilities from the second year were equal in both groups, as presented in Table 1. However, a larger proportion of the sample in the intervention group remained in more favourable health states compared to the no intervention group due to increased recovery during the first year. For example, after 2 years in hypothetical cohorts of 100 people in each group, 25 people in the intervention group remained in the mild or moderate health state compared to 22 people in the no intervention group. From years 6 to 8, the intervention also led to an effect on reduced mortality resulting in three additional life years saved in a sample of 100 persons. Overall, the intervention led to a positive accumulation of QALYs as well as reduced societal costs from year 1 to 8, see Table 4. In terms of days in full health, the QALYs gains amounted to 19 days (main analysis) or 9 days (sensitivity analysis).

**Table 4:** Studies used to estimate intervention effect in terms of recovery from bathing disability

Author, year (ref)	Sample	Follow-up	Recovery, *n* (%)	Increased probability for recovery
Chiu, 2004 (Chiu and Man 2004)	Intervention: 30 Control: 23	3 months	25 (83) 9 (39)	2.1 (bathing)
Zingmark, 2011 (Zingmark and Bernspång 2011)	Intervention: 46	3 months	32 (70)	2.8 (bathing)
	Control: 28		7 (25)	
Lewin, 2013 (Lewin et al. 2013a)	Intervention: 375	3 months	272 (73)	2.0 (personal care^a^)
	Control: 375		137(37)	
Lewin, 2013 (Lewin et al. 2013a)	Intervention: 375	1 year	308 (82)	1.4 (personal care^a^)
	Control: 375		224 (60)	
Lewin, 2014 (Lewin et al. 2014)	Intervention: 201	2 years	178 (89)	1.4 (personal care^a^)
	Control: 246		161 (65)	
Intervention characteristics				
Zingmark, 2011 (Zingmark and Bernspång 2011)	Older people who applied for home care with bathing. Interventions implemented by occupational therapists, on average 3 home visits. Focus on supporting the person to gradually increase her/his ability to safely and independently perform the tasks related to bathing. Seventy percent of the interventions focused on a modified task performance for example by the use of technical aids.			
Chiu, 2004 (Chiu and Man 2004)	Older stroke patients with an identified need of a bathing device. Additional support from occupational therapists in using prescribed assistive devices after discharge from the hospital (2–3 home visits). Interventions included demonstration, information and opportunity to practice how to use assistive devices. Information and support were given both to the older person and potential caregivers.			
Lewin, 2013 (Lewin et al. 2013a)	Older people referred to a home care service for help with personal care*. Multi-professional intervention aimed at enhancing engagement and independence in daily activities, implemented during a maximum of 12 weeks. Individually tailored intervention based on clients’ goals including, for example, use of assistive devices, exercise to enhance mobility, fall prevention, nutrition, disease self-management.			
Lewin, 2014 (Lewin et al. 2014)	Same as above.			

^a^Most common reason for personal care was bathing

### Sensitivity analysis

The sensitivity analysis showed that the size of the intervention effect was a more critical parameter than the intervention cost. When the intervention effect decreased, both QALYs gained and cost savings were reduced, but the intervention still resulted in QALY gains and was cost saving compared to no intervention. Although the intervention costs were more than 4 times as high with the multi-professional intervention, the costs for the intervention were still small compared to other societal costs.

In both the main analysis and the sensitivity analysis, the intervention resulted in more QALYs gained and lower societal costs compared to no intervention (Table 4). The intervention was cost saving, independent of time perspective and clearly dominates no intervention (Table 5).

**Table 5:** Quality-adjusted life years (QALYs) and costs at 8 years

Analysis	No intervention		Occupational therapy		Incremental QALYs	Incremental costs (€)
	QALYs^a^	Costs (€)	QALYs	Costs (€)		
Main analysis	2.211	94,982	2.263	92,572	0.052	−2410
Sensitivity analysis						
Reduced intervention effect	2.211	94,982	2.237	93,837	0.026	−1145
Increased intervention cost	2.211	94,982	2.263	92,969	0.052	−2013
Combined sensitivity analysis^b^	2.211	94,982	2.237	94,235	0.026	−747

The table shows the average accumulated QALYs and costs for one person. Incremental QALYs and costs are given for occupational therapy in relation to no intervention

^a^Quality-adjusted life years

^b^Includes a reduced intervention effect (1.2 instead of 1.4) combined with an increased intervention cost (546 € instead of 128 €)

## Discussion

This study showed that an intervention implemented to reduce bathing disability results in QALYs gained and cost savings for up to 8 years compared to no intervention. In a hypothetical cohort of 100 people with bathing disability, the intervention resulted in 5.2 QALYs gained and approximately 240,000 € in reduced societal costs. Considering that bathing disability is common among older people (Gill et al. 2006b), the intervention is clearly clinically important. For the older person who experiences bathing disability, recovery to a less dependent state leads to improved QoL both in the short and long terms. From a societal perspective, the cost related to elderly care and accommodation is substantial, and therefore an intervention that reduces these costs can contribute to allow resources to be used for the implementation of other interventions.

The results must be interpreted based on the modelling approach used. Any modelling approach is a simplification of a real-world scenario, and the validity of the results is dependent on the fit between the model and the real world (Pouryamout et al. 2012). The Markov model used in this study is based on assumptions concerning how older people transition between various states of dependency. Among older people, the onset of disability may be a dynamic process including both recovery and short periods of temporary disability (Gill et al. 2002), but over the long term, the prevalence of disability and dependency increases (Jagger et al. 2001). The Markov model was developed in close collaboration with a group of experienced social workers, and in combination with previous research on the progression of disability and dependency (Jagger et al. 2001), we conclude that our model provides a logical representation of clinically relevant states of dependency in a Swedish context. However, to further validate or refute the transition probabilities used in this study, there is a need for data on the development of dependency and mortality from longitudinal trials conducted in different contexts, e.g. different countries. In addition, transition probabilities are also likely to change over time as a consequence of public health development. Furthermore, in a modelling study, all input parameters are subject to uncertainty concerning their estimates (e.g. magnitude of intervention effect, costs and QoL) (Briggs et al. 2006) (Pouryamout et al. 2012).

### Transition probabilities

While some studies indicate that older people with limited dependency in IADL or ADL have a high probability of remaining in the same state or recover over time (Hardy and Gill 2004; Raîche et al. 2012), other studies have shown a higher risk for decline from less severe states (Nikolova et al. 2011; Pérès et al. 2005). Differences in transition probabilities likely depend on factors such as the population from which data are derived and how health states are defined. Although the existing evidence shows variation in the transition probabilities for stability and decline, several studies verify that the probability for recovery decreases in more severe states (Nikolova et al. 2011; Pérès et al. 2005; Raîche et al. 2012). We used transition probabilities based on a Canadian study. Although there may be contextual factors that impact transitions between dependency states, we have no reason to believe that there are major differences between a Canadian and a Swedish context. However, we acknowledge that further research is needed to validate transition probabilities, specifically for the context in which the model is applied in order to increase the precision of the model.

### Societal costs

Our findings are consistent with recent studies that have demonstrated that an intervention that promotes independence in ADL has a significant impact on the use and costs of health and social care (Cook et al. 2013; Lewin et al. 2013b). An especially critical estimate in focusing on recovery from severe to moderate dependency is the cost associated with each state in the model, and a large difference in costs between the two states will inevitably have a very strong impact on cost effectiveness. For the severe dependency state, we used the costs associated with dependency in 1 PADL and 2–4 IADLs (Lindholm et al. 2013). An alternative would have been to use the cost associated with dependency in 1 PADL and more than 5 IADLs, a cost estimated to be twice as high as the cost we used (Lindholm et al. 2013). We chose the more conservative cost estimate to avoid inflation of effects in terms of cost savings. However, it is clear that recovering from dependency or maintaining independence in PADL substantially impacts societal costs (Cook et al. 2013; Lewin et al. 2013b; Lindholm et al. 2013). By including informal care in the estimates of costs, Lindholm et al. acknowledge the importance of informal care. The extent of informal care is substantial and as such could have a major impact on overall cost estimates. It should be noted that the type of informal caregiver, e.g. in-home spouse of other caregiver, could have an impact on the valuation of costs. However, Lindholm et al. choose a conservative estimate of the unit costs based on the value of lost leisure time for the informal carer, approximately one-fifth of the unit cost for home help.

### Quality of life

The QALY gains estimated in this study can be considered low based on the follow-up period of 8 years, but is consistent with the findings from a review of cost–utility analysis in which the median QALY gain was 0.06 (Wisløff et al. 2014). A challenge in modelling studies is the identification of QoL scores for each health state (Pouryamout et al. 2012). We collected data from various sources (Andersen et al. 2004; Fusco et al. 2012; Honkanen et al. 2006; Szanton et al. 2011; Zingmark et al. 2014) to establish reasonable estimates of QoL scores reflecting that loss of independence negatively affects QoL (Shearer et al. 2012). Four of these studies (Andersen et al. 2004; Fusco et al. 2012; Szanton et al. 2011; Zingmark et al. 2014) used EQ-5D, which is the most commonly used instrument to estimate QoL scores (Wisløff et al. 2014), and one study used the Health Utility Index (Honkanen et al. 2006). Although different instruments may yield different QoL scores for the same health state and vary concerning their sensitivity (Fryback et al. 2010), we do not consider the use of different instruments as a major threat to the validity of the estimates of QoL scores in our study. Even with other QoL scores, as long as the score is associated with level of dependency, the intervention would result in QALY gains relative to no intervention. Since the intervention resulted in recued societal costs in both analyses, different QoL scores would not impact that the intervention was cost effective. However, to further enhance the precision of the model, we acknowledge the need to derive QoL scores for the specific population under study.

### Intervention effect and intervention cost

We identified four studies in which interventions had been implemented that were occupation-based (Fisher 2013) and focused on the performance of various tasks related to bathing. These studies (Table 3) indicated that the intervention effect was somewhat reduced over time but still impacted long-term dependency in personal care (including bathing) (Lewin et al. 2014). Our estimate of intervention effect was based on the intention to treat analysis reported by Lewin et al. (15) and can be considered a conservative estimate of intervention effect compared to the as-treated analysis in which the intervention effect was 1.5. Additional trials confirm that interventions that focus on promoting performance of and independence in IADL and PADLs are effective in improvig ADL ability (Fisher et al. 2007; Hagsten et al. 2004) and increase the probability for recovery from dependency in IADL and PADL (Cook et al. 2013).

The results also indicate that the intervention indirectly may have an impact on mortality. It is known that mortality is related to the degree of disability (Cook et al. 2013; Nikolova et al. 2011; Pérès et al. 2005), and previous research has found that interventions that focus on improving performance of ADLs have also affected mortality (Cook et al. 2013; Gitlin et al. 2009). In our study, the accumulation of QALYs is a result related both to the time spent in less severe health states and also to life years saved from year 6 to 8.

The sensitivity analysis showed that the cost for the intervention minimally impacted the overall cost effectiveness, whereas intervention effectiveness seems to have had a more significant impact. Although the results indicate that the intervention was cost saving independent of time, it is relevant to consider the content of the intervention and how it is delivered. The low intervention cost supports the notion that the intervention could be expanded if additional effects were to be obtained. The tendency that the intervention effects decline after 3 months calls for further research to explore if the initial intervention effect could be sustained. According to our knowledge, no previous trials focusing on disability in bathing or personal care have implemented interventions beyond 3 months. However, considering the low cost for the intervention, practically negligible in relation to other societal costs, any additional gain in intervention efficacy is likely to further improve cost effectiveness.

## Conclusion

This study demonstrates that an intervention that supports recovery from bathing disability is very cost effective over both the short and long term. The intervention leads to QALY gains and saves costs at any follow-up until 8 years, and thus resources can be used to implement other interventions. The most important factor for the magnitude of QALY gains and cost savings is the intervention effect. In contrast to the societal cost for elderly care and accommodation, the cost for the intervention is very small, indicating that it is worthwhile to explore if additional intervention content, such as follow-up sessions, could further enhance the intervention effect. Although our model was based on empirical evidence, we acknowledge that further refinement of the model parameters could enhance the precision of estimates of QALYs gained and cost savings.
